# Identification of Core Genes and Pathways in Melanoma Metastasis via Bioinformatics Analysis

**DOI:** 10.3390/ijms23020794

**Published:** 2022-01-12

**Authors:** Renjian Xie, Bifei Li, Lee Jia, Yumei Li

**Affiliations:** 1 Key Laboratory of Prevention and Treatment of Cardiovascular and Cerebrovascular Diseases, Ministry of Education, Gannan Medical University, Ganzhou 341000, China; RonnieWanr@gmail.com; 2 Key Laboratory of Biomaterials and Bio-Fabrication in Tissue Engineering of Jiangxi Province, Ganzhou 341000, China; 3 School of Medical Information Engineering, Gannan Medical University, Ganzhou 341000, China; 4 Institute of Oceanography, Minjiang University, Fuzhou 350108, China; 19b958029@stu.hit.edu.cn (B.L.); cmapcjia1234@163.com (L.J.); 5 School of Basic Medicine, Gannan Medical University, Ganzhou 341000, China

**Keywords:** melanoma metastasis, bioinformatics analysis, differentially expressed genes, hub gene, *KRT5*

## Abstract

Metastasis is the leading cause of melanoma-related mortality. Current therapies are rarely curative for metastatic melanoma, revealing the urgent need to identify more effective preventive and therapeutic targets. This study aimed to screen the core genes and molecular mechanisms related to melanoma metastasis. A gene expression profile, GSE8401, including 31 primary melanoma and 52 metastatic melanoma clinical samples, was downloaded from the Gene Expression Omnibus (GEO) database. The differentially expressed genes (DEGs) between melanoma metastases and primary melanoma were screened using GEO2R tool. Gene ontology (GO) and Kyoto Encyclopedia of Genes and Genome (KEGG) analyses of DEGs were performed using the Database for Annotation Visualization and Integrated Discovery (DAVID). The Search Tool for the Retrieval of Interacting Genes (STRING) and Cytoscape with Molecular Complex Detection (MCODE) plug-in tools were utilized to detect the protein–protein interaction (PPI) network among DEGs. The top 10 genes with the highest degrees of the PPI network were defined as hub genes. In the results, 425 DEGs, including 60 upregulated genes and 365 downregulated genes, were identified. The upregulated genes were enriched in ECM–receptor interactions and the regulation of actin cytoskeleton, while 365 downregulated genes were enriched in amoebiasis, melanogenesis, and ECM–receptor interactions. The defined hub genes included *CDK1*, *COL17A1*, *EGFR*, *DSG1*, *KRT14*, *FLG*, *CDH1*, *DSP*, *IVL*, and *KRT5*. In addition, the mRNA and protein levels of the hub genes during melanoma metastasis were verified in the TCGA database and paired post- and premetastatic melanoma cells, respectively. Finally, *KRT5*-specific siRNAs were utilized to reduce the *KRT5* expression in melanoma A375 cells. An MTT assay and a colony formation assay showed that *KRT5* knockdown significantly promoted the proliferation of A375 cells. A Transwell assay further suggested that *KRT5* knockdown significantly increased the cell migration and cell invasion of A375 cells. This bioinformatics study provided a deeper understanding of the molecular mechanisms of melanoma metastasis. The in vitro experiments showed that *KRT5* played the inhibitory effects on melanoma metastasis. Therefore, *KRT5* may serve important roles in melanoma metastasis.

## 1. Introduction

Melanoma, as the most common and grim malignant skin cancer, has an incidence that has unfortunately been steadily increasing in the last 40 years, and its incidence is ascending faster than that of any other solid tumor. Due to the extraordinary predisposition of melanoma to spread and its rapid progression toward metastasis, patients who develop metastasis almost always have an incurable disease, with only 6–9 months of median survival time, a 15% 3-year survival rate, and only a 4.6% 5-year survival rate [1].

Metastasis is a serious event in the clinic, leading to the majority of deaths of melanoma patients [2]. Despite the landscape of genetic alterations and elaborate molecular mechanisms discovered in melanoma, little information about the underlying biology that drives its metastasis has been fully elucidated [3]. Therefore, a more in-depth understanding of the metastatic melanoma process is urgently needed, with the aim to develop specific therapies for improving current therapy and reducing the mortality of melanoma patients.

Historically, numerous genes have been identified and are expected to be targets for preventing melanoma metastasis. For instance, *PGC1A* encodes PGC1α, which is a metabolic transcriptional coactivator and suppresses melanoma metastasis by protecting against oxidative stress [4]. The overexpression of *KISS1* inhibits the metastasis of C8161 melanoma cells [5]. *BRIC5*-encoded survivin promotes melanoma metastasis through the Akt-dependent upregulation of α5 integrin [6]. Our previous work also uncovered the oncogenic functions and regulatory mechanisms of *NOL7* and *WDR74* in melanoma [7,8]. However, these findings were hardly sufficient to develop a complete overview of melanoma metastasis. Furthermore, the peculiarity of the high heterogeneity and clinical phenotype of melanoma cells imply a complicated regulatory mechanism of cancer metastasis [9].

Microarray-based gene expression analysis is a powerful and promising tool for functional genomics research, especially for the accurate and comprehensive analysis of complex networks involved in biological processes [10,11]. In our present study, we chose the GSE8401 profile from the Gene Expression Omnibus (GEO) database and utilized the GEO2R tool to identify the differentially expressed genes (DEGs). Subsequently, gene ontology (GO) and Kyoto Encyclopedia of Genes and Genome (KEGG) pathway analyses were performed, and a protein–protein interaction (PPI) network was constructed. In general, the hub genes, including *KRT5*, *IVL*, and *DSP*, and key pathways associated with melanoma metastasis defined in the present study may provide new insights into clinical melanoma treatment. Additionally, the effects of *KRT5* on cell proliferation and cell metastatic behaviors of melanoma were determined by in vitro experiments. 

## 2. Results

### 2.1. Identification of DEGs

**Figure 1:**
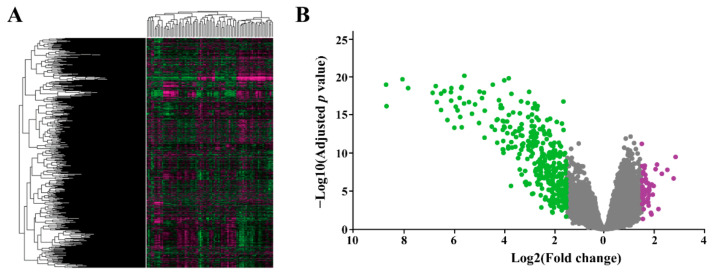
(**A**) Heat map visualization showing alternation for the full range of gene expression patterns between melanoma metastases and primary melanoma. Purple indicates upregulated genes, and green indicates downregulated genes. (**B**) The volcano plot shows the genes expressed significantly differentially between melanoma metastases and primary melanoma. Purple indicates the upregulated genes, and green indicates the downregulated genes.

The GSE8401 profile, including 31 primary melanoma samples and 52 melanoma metastasis samples, was submitted to the GEO2R online analysis tool. Using an adjusted *p* value < 0.05 and |log2 (Fold change)| > 1.5 as the cutoff criteria, a total of 425 DEGs, including 60 upregulated genes and 365 downregulated genes, were picked out. The heat map of the full range of genes and volcano plot of DEGs are shown in Figure 1. A list of DEGs is shown in Table S1. The top 15 significantly upregulated and downregulated genes are listed in Table 1. *PSPH* was the most significantly upregulated gene, and *S100A7* was the most significantly downregulated gene.

### 2.2. GO Function and KEGG Pathway Enrichment Analyses of DEGs

The top five enriched terms identified in each GO category using Database for Annotation Visualization and Integrated Discovery (DAVID) software are shown in Table S2. The upregulated genes were significantly found to participate in the formation of cellular components, including condensed chromosome kinetochore, chromosome and centromeric region; condensed chromosome and centromeric region; and condensed chromosome, chromosomal region. The biological processes included sister chromatid segregation, mitotic cell cycle process, nuclear chromosome segregation, mitotic nuclear division, and cell cycle process. The molecular functions included small molecule binding, adenyl ribonucleotide binding, adenyl nucleotide binding, ATP binding, and nucleotide binding. In addition, the downregulated genes were mainly involved in cellular components, such as the extracellular region, extracellular region part, extracellular exosome, extracellular vesicle, and extracellular organelle; biological processes including epidermis development, skin development, keratinocyte differentiation, epidermal cell differentiation, and epithelial cell differentiation; and molecular functions including structural molecule activity, endo-peptidase inhibitor activity, structural constituent of cytoskeleton, endopeptidase regulator activity, and peptidase inhibitor activity.

A KEGG pathway analysis was then conducted. As shown in Table 2, the upregulated genes were enriched in ECM-receptor interactions, progesterone-mediated oocyte maturation, regulation of actin cytoskeleton, and metabolic pathways, while the downregulated genes were enriched in amoebiasis, melanogenesis, and ECM-receptor interactions.

### 2.3. Hub Genes and Module Screening from PPI Network

**Figure 2:**
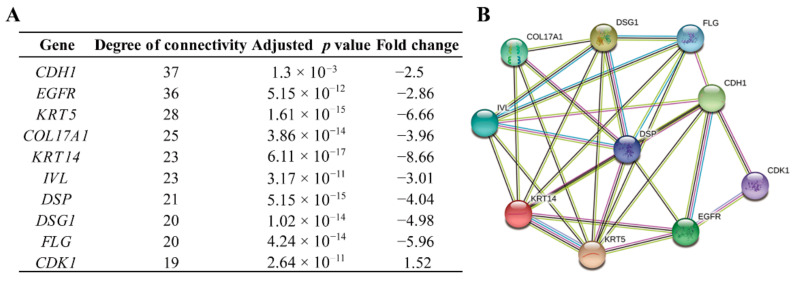
Top 10 hub genes with the highest degrees of connectivity. (**A**) Hub genes are listed. (**B**) The PPI network of the top 10 hub genes.

**Figure 3:**
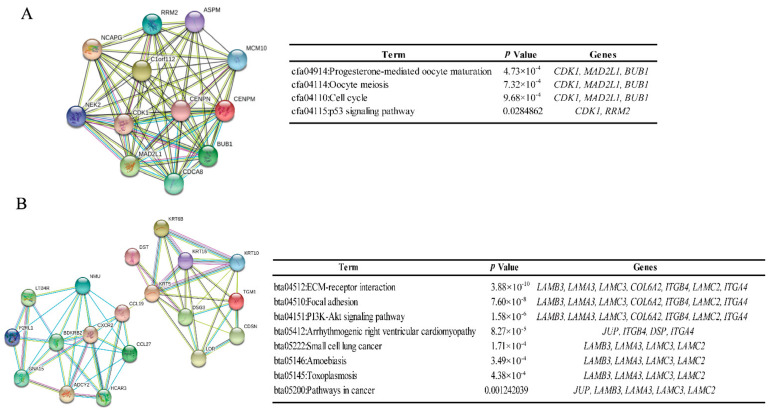
Top two modules from the PPI network. (**A**) Module 1 and the enriched pathways of module 1. (**B**) Module 2 and the enriched pathways of module 2.

Based on the information in the Search Tool for the Retrieval of Interacting Genes (STRING) protein query from the public databases of the DEGs, the top 10 genes with the highest degrees of connectivity were selected and defined as hub genes (Figure 2). Additionally, 250 nodes and 751 edges in the PPI network among those DEGs were analyzed using the Molecular Complex Detection (MCODE) plug-in in Cytoscape, and the top 2 significant modules were filtered (Figure 3). Based on the GO function and KEGG pathway analyses, these two modules were principally associated with cell cycle, ECM-receptor interactions, focal adhesion, and the PI3K-Akt signaling pathway. 

### 2.4. Validation of Hub Gene Expression between Primary Melanoma and Metastatic Site in TCGA Database

**Figure 4:**
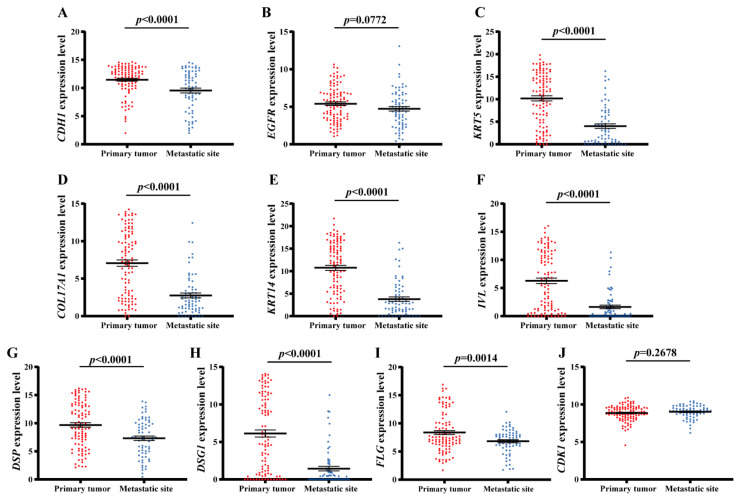
Expression levels of hub genes in melanoma metastases (*n* = 71) and primary melanoma (*n* = 105) visualized through the TCGA database. Histograms represents the expression levels of (**A**) *CDH1*, (**B**) *EGFR*, (**C**) *KRT5*, (**D**) *COL17A1*, (**E**) *KRT14*, (**F**) *IVL*, (**G**) *DSP*, (**H**) *DSG1*, (**I**) *FLG*, and (**J**) *CDK1*. *p* value < 0.05 was considered significant, *t*-test.

To confirm the reliability and accuracy of the results through the above bioinformatics analysis, we verified the mRNA level of the hub genes in the TCGA database. As shown in Figure 4, The results showed that gene expression levels of *COL17A1*, *DSG1*, *KRT14*, *FLG*, *CDH1*, *DSP*, *IVL*, and *KRT5* had significant decreases in metastatic melanoma (*n* = 71) compared with primary melanoma (*n* = 105). However, the gene expression level of *CDK1* and *EGFR* had no significant differences between metastatic melanoma and primary melanoma.

### 2.5. Validation of Protein Expression of Hub Genes in Paired Premetastatic and Postmetastatic Melanoma Cells

**Figure 5:**
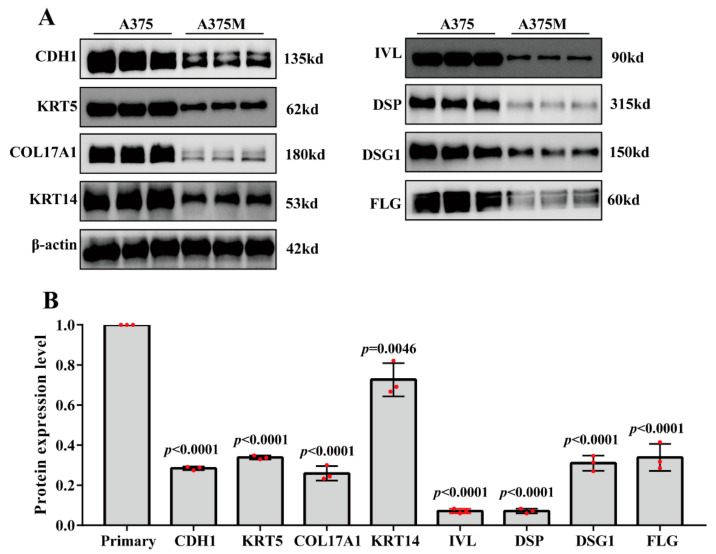
Western blotting staining (**A**) and quantitative analysis (**B**) of CDH1, KRT5, COL17A1, KRT14, IVL, DSP, DSG1, and FLG between metastatic melanoma cells and primary melanoma cells. β-actin served as the internal control. Data are means ± s.d., *n* ≥ 3; *p* value < 0.05 was considered significant, *t*-test.

Based on the above data (Figure 4), the gene expressions of *CDH1*, *KRT5*, *COL17A1*, *KRT14*, *IVL*, *DSP*, *DSG1*, and *FLG* show the consistent downregulation in metastatic melanoma compared with primary melanoma. To further confirm the protein expression levels of these eight hub genes in the development of melanoma metastasis, paired premetastatic melanoma cells (A375) and postmetastatic melanoma cells (A375M) were established through an experimental animal model of melanoma metastasis (see the Materials and Methods section). Next, western blotting was carried out to measure the protein level of those eight hub genes. As shown in Figure 5, the expression of these eight hub genes was significantly downregulated in metastatic melanoma cells compared with the primary melanoma cells (*p* < 0.01).

### 2.6. KRT5 Knockdown Promotes Cell Proliferation, Migration, and Invasion of Melanoma

**Figure 6:**
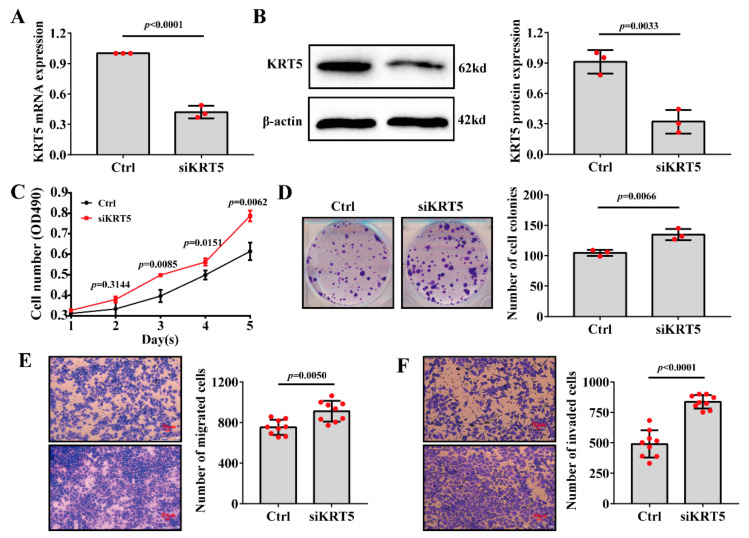
*KRT5* knockdown promoted the proliferation and metastasis of A375 cells. (**A**,**B**), Successes of *KRT5* knockdown in A375 cells following transfection with the *KRT5*-specific-siRNAs, as examined by qRT-PCR assay (**A**) and western blotting (**B**). (**C**) *KRT5* knockdown accelerated cell proliferation of A375 cells, as examined by MTT assay. (**D**) *KRT5* knockdown promoted cell colony formation of A375 cells, as detected by cell colony formation assay. (**E**,**F**) KRT5 knockdown enhanced the cell migration (**E**) and invasion (**F**) of A375 cells, as detected by Transwell assay. Data are means ± s.d., *n* ≥ 3; *p* value < 0.05 was considered significant, *t*-test.

To further prove the reliability and accuracy of this bioinformatics analysis, one of the hub gene, *KRT5*, was selected for further biological experiments analysis. The reduction efficiency was determined by siRNA knockdown of *KRT5*, followed by RT-qPCR and western blotting (Figure 6A,B). The MTT experiments demonstrated that the proliferative rate of A375 cells was significantly increased upon *KRT5* knockdown (Figure 6C). The colony formation experiments revealed that the number of colonies in the *KRT5*-siRNA transfection group was significantly higher compared with that in the control group (Figure 6D). The Transwell assay indicated that the migration and invasion abilities were significantly enhanced following *KRT5* knockdown in A375 cells (Figure 6E,F). These results suggested the inhibitory effect of *KRT5* in melanoma metastasis.

## 3. Discussion

Melanoma is the most malignant type of skin cancer, and its metastasis remains essentially incurable because the mutated genes and underlying molecular mechanisms are poorly uncovered [12]. In the present study, the gene expression profile of GSE8401 was analyzed, and 425 DEGs, including 60 upregulated genes and 365 downregulated genes, were filtered between 31 primary melanoma samples and 52 metastatic melanoma samples, which were obtained from the clinical melanoma patients.

The GO function analysis revealed that upregulated DEGs mainly participated in small molecule binding, nucleotide binding, and cell cycle processes, while the downregulated DEGs were involved in extracellular region, extracellular region part, extracellular organelle, exosome, and vesicles. Based on these results, cell mitosis and malignant proliferation were activated, whereas the interaction with the extracellular environment was suppressed during the metastatic process.

The KEGG pathway analysis revealed that upregulated DEGs were mainly enriched in ECM–receptor interactions, progesterone-mediated oocyte maturation, regulation of the actin cytoskeleton, and metabolic pathways and that downregulated DEGs were enriched in amoebiasis, melanogenesis, and ECM-receptor interactions. Both upregulated genes and downregulated genes were enriched in ECM-receptor interactions, indicating that this pathway is pronouncedly valuable to melanoma metastasis. Cross-talk between the ECM and melanoma metastasis is well known to be commonly elaborate in previous reports [13,14]. Cancer metastasis involves multiple complex processes that are critically influenced by ECM components [15]. Various ECM-related proteins are significantly dysregulated during the progression of cancer, causing both biochemical and biomechanical changes that together promote cancer metastasis [16]. Herein, the expressions of *ITGA4*, *SPP1*, and *ITGB3* were increased, while the *COL6A2*, *LAMC2*, *LAMB3*, *SDC1*, *ITGB4*, *LAMC3*, *COMP*, and *LAMA3* expressions were decreased. It prompted the genes *ITGA4*, *SPP1*, and *ITGB3* to promote the interactions between melanoma cells and the ECM and thus facilitated melanoma metastasis. On the contrary, the genes *COL6A2*, *LAMC2*, *LAMB3*, *SDC1*, *ITGB4*, *LAMC3*, *COMP*, and *LAMA3* might play opposite effects since the regulation of actin cytoskeleton, metabolic pathways, and melanogenesis are the key pathways involved in melanoma metastasis [17,18,19]. The regulation of action cytoskeleton is mainly related to cell migration. The dynamic actin cytoskeleton spatially and temporally mediates protrusion, adhesion, contraction, and retraction from the active cell. The variability of action cytoskeleton of cancer cells confers the aggressive phenotype, such as the EMT. Several genes, such as *FAK* and *c-fos*, were attested to regulate the actin cytoskeleton to promote melanoma metastasis [20,21]. Dysregulation of metabolic pathways in cancer cells results in the metabolic reprogramming in cancer, leading to enhanced substance uptake to supply the energy production and biosynthesis. It was declared that metabolic remodeling is pivotal for melanoma cells to adapt to tumor microenvironment and to maintain the growth and dissemination of melanoma cells [22]. Obviously, melanogenesis is essential for melanoma development. Deregulated melanogenesis potentially contributes to more aggressive behaviors of melanoma cells [23].

Direct evidence of the participation of progesterone-mediated oocyte maturation and amoebiasis in melanoma metastasis has so far not been illustrated. Rare studies in the literature have shown the interaction between cancer metastasis and progesterone-mediated oocyte maturation/amoebiasis. Mood et al. showed that progesterone-mediated Xenopus oocytes maturation pathway was related to the G2/M transition of oocytes [24]. In the current study, *CDK1*, *MAD2L1*, and *BUB1*, which are associated with the cell cycle, were identified to enrich progesterone-mediated oocyte maturation. This implies that the progesterone-mediated oocyte maturation pathway might participate in melanoma metastasis by influencing the cell cycle. In addition, Nelis et al. presented that cancer cells and amebic trophozoites follow the same metastatic route for the liver and other organs [25]. Cancer-related genes that are adhesion/migration-related genes, including *LAMB3*, *LAMA3*, *LAMC2*, and *LAMC3*, and immune-related genes, including *SERPINB3*, *SERPINB4*, and *IL1R2*, as well as the proliferation-related genes, including *ARG1* and *SERPINB2*, which were enriched in the amoebiasis pathway in this analysis. This implies that the amoebiasis pathway might be involved in melanoma metastasis through these genes. These pathways screened in this study provided promising targets for new drug intervention to fight melanoma.

Most importantly, 10 hub genes were defined. We verified the mRNA expression of those hub genes in various samples of primary melanoma and metastatic melanoma, which were obtained from the TCGA dataset. The overall direction in the TCGA dataset yielded concordance with the results of bioinformatics analysis upon GSE8401, except for *EGFR* and *CDK1*. It appears that *CDH1*, *KRT5*, *COL17A1*, *KRT14*, *IVL*, *DSP*, *DSG1*, and *FLG* may be potentially useful biomarkers in melanoma metastasis. To further confirm this hypothesis, we tested the protein levels of these eight genes in paired metastatic melanoma cells and primary melanoma cells. Consistently, the protein expression levels of *CDH1*, *KRT5*, *COL17A1*, *KRT14*, *IVL*, *DSP*, *DSG1*, and *FLG* were significantly downregulated in metastatic melanoma compared with primary melanoma. 

Among these hub genes, we identified the attenuated expressions of *CDH1*, *COL17A1*, *DSG1*, *KRT14*, and *FLG* in melanoma metastases compared with primary melanoma via bioinformatics analysis upon the GSE8401 and TCGA datasets. This implies that these genes may suppress melanoma metastasis. There have been several articles that revealed the expression pattern and functional role of those genes in melanoma development. *CDH1* encodes E-cadherin, a key biomarker of epithelial–mesenchymal transition (EMT) in cancer cells and a mediator of cell–cell adhesion in epithelial tissues [26]. Loss of E-cadherin promotes the aggressive behaviors of melanoma cells via constitutively active snail expression during the metastasis process [20,27,28]. *COL17A1* encodes the collagen alpha-1 (XVII) chain, which is responsible for the adhesion of cells and matrix. Kai Tao et al. found that *COL17A1* was decreased in melanoma compared with normal tissues through bioinformatics analysis, while an inverse result was observed in the qRT-PCR validation [29]. *DSG1*-encoded desmoglein 1 is the component of intercellular desmosome junctions and is involved in cell–cell adhesion [30]. Ji et al. declared that *DSG1* might have a potential value for the prognosis and treatment of melanoma, and Herlyn et al. detected that inhibiting *DSG1* contributed to melanoma metastasis [31,32]. *KRT14* encodes keratin 14, which is an epithelial proliferative marker [33]. It was reported that *KRT14* potentially facilitates melanoma tumorigenesis [34,35]. *FLG* encodes Filaggrin, which is a functional protein in the epidermis and plays a critical role in skin homeostasis. *FLG* was observed to participate in melanoma development [31,36]. 

It is noticeable that the functional characteristics of *KRT5*, *IVL*, and *DSP* in melanoma have not been fully explored, especially in melanoma metastasis. *KRT5* encodes keratin 5, which belongs to the keratin family, which are intermediate filament proteins. Keratin 5 is responsible for the structural integrity of epithelial cells and contributes to cell polarization, cytoskeleton regulation, and protein translation [37]. Previous research has confirmed that keratin 5 is a stem cell marker in breast cancer and is associated with cancer recurrence and chemotherapy resistance in ovarian cancer [37,38]. *IVL* encoding the involucrin is a marker of keratinocyte terminal differentiation and maintains the morphological characteristics of the epidermis [39]. Previous findings have suggested that involucrin is a biomarker of EMT in squamous cell carcinoma [40]. Another group also demonstrated that involucrin might be involved in breast cancer, cervical cancer, and oral cancer [41,42]. *DSP* encodes desmoplakin, which is a major high molecular weight protein of desmosomes. It was shown that *DSP* functions as a tumor suppressor in cancer migration [43,44], whereas researchers also indicated that the deficiency of desmoplakin induced loss of the epithelial phenotype and acquisition of aggressive phenotype and thus facilitated melanoma metastasis [45]. The explicit function and regulatory mechanism of *DSP* in melanoma metastasis are not yet understood. 

In the present study, the downregulation of the *KRT5*, *IVL*, and *DSP* expressions implied inhibitory effects of these genes in melanoma metastasis. To further test this hypothesis, *KRT5* was selected for further biological analysis as there are few studies on *KRT5* in the literature and the mechanism related to melanoma metastasis is unclear. We suppressed the *KRT* expression in melanoma A375 cells through *KRT5*-specfic siRNAs and then performed the in vitro experiments to clarify the effects of *KRT5* on cell proliferation, migration, and invasion of melanoma. The experimental results showed that *KRT5* knockdown can significantly enhance the cell metastasis capacities in melanoma, including cell proliferation, migration, and invasion. 

In this study, we highlighted that *KRT5*, *IVL*, and *DSP* may serve as novel targets to suppress melanoma metastasis. The possible effect of *KRT5* on melanoma metastasis were preliminarily investigated through in vitro biological experiments. Further studies about the detailed biological function and regulatory mechanisms of these genes in melanoma metastasis are needed in the future.

## 4. Materials and Methods

### 4.1. Microarray Data

The gene expression dataset GSE8401 (GDS3966) (https://www.ncbi.nlm.nih.gov/geo/query/acc.cgi?acc=GSE8401, 3 January 2022), based on the Agilent GPL96 platform ((HG-U133A) Affymetrix Human Genome U133A Array), was obtained from GEO, a free and publicly available database [46]. The GSE8401 dataset includes 83 clinical samples, containing 31 primary melanomas and 52 melanoma metastases. The heat map of the full expression range of genes was acquired using the GEO online data analysis tool (https://www.ncbi.nlm.nih.gov/sites/GDSbrowser, 3 January 2022). The volcano plot of the full expression range of genes was established using Microsoft Excel.

### 4.2. Identification of the DEGs

GEO2R (https://www.ncbi.nlm.nih.gov/geo/geo2r/, 3 January 2022), an interactive web tool, allows users to compare different groups of clinical cancer samples in a GEO series to identify genes that were differentially expressed across different experimental conditions [47]. We applied this tool to detect the DEGs between primary melanomas and melanoma metastases in GSE8410. The results are presented in a table of genes ordered by significance. The adjusted *p*-value was calculated to reduce the false positive rate, applying the Benjamini and Hochberg false discovery rate method by default. Genes within the cutoff criteria of an adjusted *p*-value < 0.05 and |log2 (Fold change)| ≥ 1.5 were designated DEGs. 

### 4.3. GO Function and KEGG Pathway Analyses of DEGs

Gene ontology (GO) analysis is a commonly used and productive method for annotating genes and gene products and for identifying the biological characteristics of high-throughput genome or transcriptome data [48]. The Kyoto Encyclopedia of Genes and Genomes (KEGG) is an open and collective database integrating genomes, biological pathways, diseases, drugs, and chemical substances. DEGs were subjected to the Database for Annotation Visualization and Integrated Discovery (DAVID, https://david.ncifcrf.gov/, 3 January 2022), an online bioinformatics tool, to interpret the GO functions and enriched KEGG pathways and to visualize the biological processes (BP), molecular functions (MF), cellular components (CC) and pathways of those DEGs [49]. A *p*-value < 0.05 and FDR < 0.05 were set as the cutoff criteria.

### 4.4. Construction of PPI Network and Module Analysis

The Search Tool for the Retrieval of Interacting Genes (STRING) is an online tool that assesses protein–protein interaction (PPI) network information [50]. STRING (version 10.5) was used to evaluate the potential PPI relationships among those DEGs. Only experimentally validated interactions with a combined score ≥0.4 were selected as significance. The PPI network was constructed and visualized using Cytoscape software 3.6.0 [51]. The molecular complex detection (MCODE) plug-in in Cytoscape was used to screen the modules of the PPI network. The inferred modules used the default settings with the degree cutoff = 2, node score cutoff = 0.2, K-core = 2, and max depth = 100. Additionally, the hub genes were mapped into STRING with a confidence score ≥0.4 and a maximum number of interactors ≤ 5. The KEGG pathway analysis of the genes in each module was performed using DAVID.

### 4.5. Definitions of Hub Genes

Based on the information in the STRING protein query and degree analysis of the PPI among DEGs using Cytoscope software, the top 10 genes with the highest degrees were defined as hub genes. 

### 4.6. Validation of Hub Gene Expression in TCGA Database

The expression level of identified hub genes between primary melanoma and metastatic site was validated using TCGA data, which contains 105 primary melanomas and 71 metastatic melanomas (https://tcga-data.nci.nih.gov/tcga/, 3 January 2022). The comparison between the two datasets was performed with the *t*-test. A *p*-value < 0.05 was considered significant.

### 4.7. Cell, Cell Culture, and Postmetastatic Cell Line Establishment

The human melanoma A375 cell line was purchased from the Cell Resource Center of Shanghai Institute for Biological Sciences (Chinese Academy of Sciences, Shanghai, China) and used as the premetastatic parental cell line. The post-metastatic melanoma cell line, A375M, was derived from the pulmonary metastatic nodules of pre-metastasized parental A375 cells via trypsinization as described previously [7,52]. Briefly, A375 cells (3 × 10^5^ cells in 0.1 mL saline solution per mouse) were intravenously injected into the tails of 6–8-week-old BALB/C nude mice (Slac Animal Inc., Shanghai, China). After 7 weeks, all mice were sacrificed, and the pulmonary metastatic nodules were stripped to obtain monoplast via trypsinization. Subsequently, these monoplasts were cultured in vitro to establish the A375M cell line. 

The cell lines mentioned above were cultured in RPMI 1640 medium (HyClone, Logan, UT, USA) supplemented with 10% (*v*/*v*) fetal bovine serum (FBS, Gemini, West Sacramento, CA, USA) and 100 U/mL penicillin/streptomycin (Sigma-Aldrich, St. Louis, MO, USA) and maintained in a humidified atmosphere with 5% CO_2_ at 37 °C. The cells were harvested by digestion with 0.25% trypsin (HyClone, Logan, UT, USA) before use.

All cell lines were regularly subjected to mycoplasma testing. The A375 and A375M cell lines were characterized by Genetic Testing Biotechnology Corporation (Suzhou, China) using short tandem repeat (STR) markers. The animal studies were performed following the animal protocol and procedures approved by the Institutional Animal Care and Use Committee (IACUC) of Fuzhou University. The identification code of this animal study is protocol #2019-SG-014, which are consistent with the AAALAS guidelines. All possible efforts were made to minimize animal suffering and sacrifice.

### 4.8. siRNA-Mediated Knockdown of KRT5 in A375 Cells

A375M cells at 70–80% confluency were transfected in OPTI-MEM medium (Invitrogen, Carlsbad, CA, USA) with the indicated siRNA duplexes using lipofectamine 3000 (Invitrogen, Carlsbad, CA, USA). After 6 h of incubation, the transfection medium was removed. Cells were washed with phosphate-buffered solution (PBS) and then cultured in complete medium for 24 to 48 h before further experiments. The reduction efficiency of *KRT5* in A375M cells was detected by RT-qPCR and western blotting. The *KRT5*-specific siRNA duplexes were synthesized by Sangon Biotech (Shanghai, China), and the sequences were listed as follows, 5′-3′: CAUCUCUGUUGUCACAAGCAGUGUU.

### 4.9. qRT-PCR

RT-qPCR was carried out as reported previously [53]. mRNA level of target genes was determined by the 2^−^^△△^^Ct^ method and normalized to *ACTB*, which was served as the internal control. Primers corresponding to indicated genes were obtained from Sangon Biotech (Shanghai, China), and listed as follows: human *KRT5* (forward: 5′-3′, AGAAGCCGAGTCCTGGTATCAGAC, reverse: 5′-3′, CTTGGTGTTGCGGAGGTCATCG), human *ACTB* (forward: 5′-3′, AGAAAATCTGGCACCACACC, reverse: 5′-3′, AGAGGCGTACAGGGATAGCA).

### 4.10. Quantification of Protein Profiles Using Western Blotting 

Protein lysates were prepared in RIPA lysis buffer, separated by SDS-PAGE, and transferred onto PVDF (polyvinylidene difluoride) membrane (Bio-Rad, Hercules, CA, USA). Membranes were probed overnight at 4 °C with the following primary antibodies at a dilution of 1:1000 unless otherwise stated: anti-COL17A1 (Abclonal, A4808, Wuhan, China), anti-DSG1 (ABclonal, A9812, Wuhan, China), anti-KRT14 (ABclonal, A19039, Wuhan, China), anti-FLG (Wanlei, WL02131, Shenyang, China), anti-CDH1 (Cell Signaling Technologies, #3195, Beverly, MA, USA), anti-DSP (Abclonal, A13299), anti-IVL (ABclonal, A13311), anti-KRT5 (Abclonal, A11396, Wuhan, China), and anti-β-actin (WanleiBio, WL01372, Shenyang, China). Then, the membranes were incubated with appropriate horseradish peroxidase–conjugated secondary antibodies (#IH0011, #IH0031, Dingguochangsheng Bio, Beijing, China). Protein bands were visualized with the hypersensitive chemiluminescence kit (Wanleibio, WLA006, Shenyang, China). Immunodetection was accomplished using a ChemiDoc XRS system (Bio-Rad, Hercules, CA, USA). Densitometric analyses were conducted using ImageLab software (Bio-Rad, Hercules, CA, USA).

### 4.11. Cell Proliferation Assay

For cell proliferative rate assay, target cells were plated in 96-well plates (5000 cells per well). Then, cells were incubated with MTT solution (JT343, Dingguochangsheng Bio, Beijing, China) for 4 h, and absorbance at 490 nm was measured by a microplate reader (Tecan Infifinite1 200Pro, Hombrechtikon, Switzerland) daily for 5 days. For cell colony formation assay, target cells (10,000 cells/well) were plated in 6-well plates and cultured for 14 days. Cell colonies were staining with crystal violet solution (DC079, Dingguochangsheng Bio, Beijing, China) and quantified by a Canon scanner (Shanghai, China).

### 4.12. Transwell Assay

Transwell assay was performed as described previously with minor modifications [8,54]. Target cells were suspended in culture medium containing 1% BSA but without FBS. These cells (10^5^ cells/200 μL culture medium per group) were plated into the top chamber of Transwell chambers (for migration) or chamber coated with Matrigel (Corning, NY, USA, for invasion). A total of 800 μL culture mediums containing 20% FBS was added into the lower chamber of Transwell chambers. Twenty-four hours later, the number of migrated or invaded cells was evaluated.

### 4.13. Statistical Analysis

The data for all experiments were managed using GraphPad Prism software 8.0 and are represented as the means ± s.d. A paired *t*-test was used for two-group comparisons. A *p*-value < 0.05 was considered statistically significant.

## 5. Conclusions

Collectively, our bioinformatics analysis presented here identified the DEGs and hub genes involved in melanoma metastasis, which might have important roles in melanoma metastasis. A total of 425 DEGs and 10 hub genes were defined, and the enrichment analysis suggested that an interaction with ECM may play a dominant role in melanoma metastasis. Overall, *KRT5*, *IVL*, and *DSP* might represent potential functions for the prevention and treatment of melanoma metastasis. Notably, *KRT5* was confirmed to play inhibitory effect on melanoma metastasis. The findings of this study may contribute to the more profound elucidation of mechanisms of melanoma metastasis. However, further verification experiments are necessary to confirm the results of these analyses.

## Figures and Tables

**Table 1:** Selected DEGs between primary melanoma and metastatic melanoma.

A. Top 15 Upregulated DEGs			
Gene Symbol	Gene ID	Log2 (Fold Change)	Adjusted *p* Value
*PSPH*	5723	2.8486465	2.98 × 10^−10^
*SPP1*	6696	2.767764	1.99 × 10^−7^
*IGF2BP3*	10643	2.5198993	1.44 × 10^−8^
*DNAJB9*	4189	2.2987063	4.92 × 10^−8^
*MAGEA6*	4105	2.1640936	2.14 × 10^−3^
*MAGEA3*	4102	2.1640936	2.14 × 10^−3^
*ADAM12*	8038	2.1087045	2.97 × 10^−9^
*RRM2*	6241	1.9764716	1.95 × 10^−6^
*ITGB3*	3690	1.9427608	2.67 × 10^−5^
*DHFR*	1719	1.8997797	1.24 × 10^−6^
*PAEP*	5047	1.8672555	6.61 × 10^−3^
*CDK1*	983	1.8363552	1.61 × 10^−6^
*UGT8*	7368	1.8051681	6.63 × 10^−6^
*EXOC5*	10640	1.7962246	3.13 × 10^−6^
*CENPN*	55839	1.7951241	3.07 × 10^−5^
**B. Top 15 Downregulated DEGs**			
**Gene Symbol**	**Gene ID**	**Log2 (** **Fold Change)**	**Adjusted *p* Value**
*S100A7*	6278	−8.6805712	9.06 × 10^−20^
*KRT14*	3861	−8.6624659	6.11 × 10^−17^
*KRT16*	3868	−8.0258657	1.74 × 10^−20^
*SPRR1A*	6698	−7.8002823	2.57 × 10^−19^
*KRT6A*	3853	−6.8262439	1.06 × 10^−18^
*KRT17*	3872	−6.7016996	1.41 × 10^−19^
*JUP*	3728	−6.7016996	1.41 × 10^−19^
*KRT5*	3852	−6.6593224	1.79 × 10^−17^
*KRT6C*	286887	−6.4903185	1.36 × 10^−18^
*KRT6B*	3854	−6.4903185	1.36 × 10^−18^
*LOR*	4014	−6.4902197	1.94 × 10^−16^
*SFN*	2810	−6.3583246	6.30 × 10^−19^
*LGALS7B*	653499	−6.2382627	4.02 × 10^−15^
*LGALS7*	3963	−6.2382627	4.02 × 10^−15^
*PKP1*	5317	−6.0861137	1.14 × 10^−17^

**Table 2:** KEGG pathway analysis of DEGs associated with melanoma metastasis.

A. Upregulated DEGs					
Category	Term	Count	*p* Value	Genes	FDR
KEGG_PATHWAY	hsa04512: ECM-receptor interaction	3	3.90 × 10^−^^1^^2^	*ITGA4*, *ITGB3*, *SPP1*	1.04 × 10^−44^
KEGG_PATHWAY	hsa04914: Progesterone-mediated oocyte maturation	3	3.92 × 10^−^^7^	*CDK1*, *MAD2L1*, *BUB1*	1.66 × 10^−39^
KEGG_PATHWAY	hsa04810: Regulation of actin cytoskeleton	4	3.94 × 10^−2^	*LIMK1*, *PIP5K1A*, *ITGA4*, *ITGB3*	5.14 × 10^−33^
KEGG_PATHWAY	hsa01100: Metabolic pathways	9	6.17 × 10^−2^	*DHFR*, *SLC33A1*, *GLUD2*, *RRM2*, *UGT8*, *PIP5K1A*, *PSPH*, *ACSL3*, *PYGB*	5.59 × 10^−19^
**B. Downregulated DEGs**					
KEGG_PATHWAY	hsa05146: Amoebiasis	12	2.00 × 10^−7^	*IL1R2*, *GNA15*, *ARG1*, *GNAL*, *LAMB3*, *LAMA3*, *LAMC3*, *SERPINB2*, *LAMC2*, *SERPINB4*, *SERPINB3*, *SERPINB13*	2.45 × 10^−^^2^^4^
KEGG_PATHWAY	hsa04916: Melanogenesis	9	2.86 × 10^−4^	*DCT*, *WNT5A*, *TYRP1*, *WNT4*, *FZD10*, *ADCY2*, *CALML3*, *EDN1*, *CALML5*	1.67 × 10^−17^
KEGG_PATHWAY	hsa04512: ECM-receptor interaction	8	6.70 × 10^−4^	*SDC1*, *LAMB3*, *LAMA3*, *LAMC3*, *COMP*, *COL6A2*, *ITGB4*, *LAMC2*	5.78 × 10^−9^

## Data Availability

Please contact the author for data requests.

## Supplementary Materials

The following supporting information can be downloaded at: https://www.mdpi.com/article/10.3390/ijms23020794/s1.

## References

[1] Eggermont A.M., Spatz A., Robert C. (2014). Cutaneous melanoma. Lancet.

[2] Braeuer R.R., Watson I.R., Wu C.J., Mobley A.K., Kamiya T., Shoshan E., Bar-Eli M. (2014). Why is melanoma so metastatic?. Pigment. Cell Melanoma Res..

[3] Owens B. (2014). Melanoma. Nature.

[4] Luo C., Lim J.-H., Lee Y., Granter S.R., Thomas A., Vazquez F., Widlund H.R., Puigserver P. (2016). A PGC1α-mediated transcriptional axis suppresses melanoma metastasis. Nature.

[5] Lee J.-H., Miele M.E., Hicks D.J., Philips K.K., Trent J.M., Weissman B.E., Welch D.R. (1996). KiSS-1, a Novel Human Malignant Melanoma Metastasis-Suppressor Gene. J. Natl. Cancer Inst..

[6] McKenzie J.A., Liu T., Jung J.Y., Jones B.B., Ekiz H.A., Welm A.L., Grossman D. (2013). Survivin promotion of melanoma metastasis requires upregulation of alpha5 integrin. Carcinogenesis.

[7] Li Y., Zhou Y., Li B., Chen F., Shen W., Lu Y., Zhong C., Zhang C., Xie H., Katanaev V.L. (2020). WDR74 modulates melanoma tumorigenesis and metastasis through the RPL5-MDM2-p53 pathway. Oncogene.

[8] Li Y., Zhong C., Wang J., Chen F., Shen W., Li B., Zheng N., Lu Y., Katanaev V.L., Jia L. (2021). NOL7 facilitates melanoma progression and metastasis. Signal Transduct. Target. Ther..

[9] Krepler C., Sproesser K., Brafford P., Beqiri M., Garman B., Xiao M., Shannan B., Watters A., Perego M., Zhang G. (2017). A comprehensive patient-derived xenograft collection representing the heterogeneity of melanoma. Cell Rep..

[10] Yan J., Huang Q. (2012). Genomics screens for metastasis genes. Cancer Metastasis Rev..

[11] Sun C., Yuan Q., Wu D., Meng X., Wang B. (2017). Identification of core genes and outcome in gastric cancer using bioinformatics analysis. Oncotarget.

[12] Siegel R.L., Miller K.D., Jemal A. (2016). Cancer statistics, 2016. CA A Cancer J. Clin..

[13] Yuzhalin A.E., Lim S.Y., Kutikhin A.G., Gordon-Weeks A.N. (2018). Dynamic matrisome: ECM remodeling factors licensing cancer progression and metastasis. Biochim. Biophys. Acta-Rev. Cancer.

[14] Schomberg J., Wang Z., Farhat A., Guo K.L., Xie J., Zhou Z., Liu J., Kovacs B., Liu-Smith F. (2020). Luteolin inhibits melanoma growth in vitro and in vivo via regulating ECM and oncogenic pathways but not ROS. Biochem. Pharmacol..

[15] Barney L.E., Dandley E.C., Jansen L.E., Reich N.G., Mercurio A., Peyton S. (2015). A cell–ECM screening method to predict breast cancer metastasis. Integr. Biol..

[16] Venning F.A., Wullkopf L., Erler J.T. (2015). Targeting ECM disrupts cancer progression. Front. Oncol..

[17] Hall A. (2009). The cytoskeleton and cancer. Cancer Metastasis Rev..

[18] Ruocco M.R., Avagliano A., Granato G., Vigliar E., Masone S., Montagnani S., Arcucci A. (2019). Metabolic flexibility in melanoma: A potential therapeutic target. Semin. Cancer Biol..

[19] Kleszczynski K., Kim T.K., Bilska B., Sarna M., Mokrzynski K., Stegemann A., Pyza E., Reiter R.J., Steinbrink K., Bohm M. (2019). Melatonin exerts oncostatic capacity and decreases melanogenesis in human MNT-1 melanoma cells. J. Pineal Res..

[20] Rich J.N. (2007). Cancer stem cells in radiation resistance. Cancer Res..

[21] Oliva I.B., Coelho R.M., Barcellos G.G., Saldanha-Gama R., Wermelinger L.S., Marcinkiewicz C., Benedeta Zingali R., Barja-Fidalgo C. (2007). Effect of RGD-disintegrins on melanoma cell growth and metastasis: Involvement of the actin cytoskeleton, FAK and c-Fos. Toxicon.

[22] Pellerin L., Carrie L., Dufau C., Nieto L., Segui B., Levade T., Riond J., Andrieu-Abadie N. (2020). Lipid metabolic Reprogramming: Role in Melanoma Progression and Therapeutic Perspectives. Cancers.

[23] Brozyna A.A., Jozwicki W., Carlson J.A., Slominski A.T. (2013). Melanogenesis affects overall and disease-free survival in patients with stage III and IV melanoma. Hum. Pathol..

[24] Mood K., Bong Y.-S., Lee H.-S., Ishimura A., Daar I.O. (2004). Contribution of JNK, Mek, Mos and PI-3K signaling to GVBD in Xenopus oocytes. Cell. Signal..

[25] Leroy A., Mareel M., De Bruyne G., Bailey G., Nelis H. (1994). Metastasis of Entamoeba histolytica compared to colon cancer: One more step in invasion. Invasion Metastasis.

[26] Nawijn M.C., Hackett T.L., Postma D.S., van Oosterhout A.J., Heijink I.H. (2011). E-cadherin: Gatekeeper of airway mucosa and allergic sensitization. Trends Immunol..

[27] Canel M., Serrels A., Frame M.C., Brunton V.G. (2013). E-cadherin–integrin crosstalk in cancer invasion and metastasis. J. Cell Sci..

[28] Kuphal S., Bosserhoff A.K. (2012). E-cadherin cell–cell communication in melanogenesis and during development of malignant melanoma. Arch. Biochem. Biophys..

[29] Zhang Q., Wang Y., Liang J., Tian Y., Zhang Y., Tao K. (2017). Bioinformatics analysis to identify the critical genes, microRNAs and long noncoding RNAs in melanoma. Medicine.

[30] Mathur M., Goodwin L., Cowin P. (1994). Interactions of the cytoplasmic domain of the desmosomal cadherin Dsg1 with plakoglobin. J. Biol. Chem..

[31] Han Y., Li X., Yan J., Ma C., Wang X., Pan H., Zheng X., Zhang Z., Gao B., Ji X.Y. (2020). Bioinformatic Analysis Identifies Potential Key Genes in the Pathogenesis of Melanoma. Front. Oncol..

[32] Li G., Schaider H., Satyamoorthy K., Hanakawa Y., Hashimoto K., Herlyn M. (2001). Downregulation of E-cadherin and Desmoglein 1 by autocrine hepaocyte growth factor during melanoma development. Oncogene.

[33] Bilandzic M., Rainczuk A., Green E., Fairweather N., Jobling T.W., Plebanski M., Stephens A.N. (2019). Keratin-14 (KRT14) Positive Leader Cells Mediate Mesothelial Clearance and Invasion by Ovarian Cancer Cells. Cancers.

[34] Wang L.X., Li Y., Chen G.Z. (2018). Network-based co-expression analysis for exploring the potential diagnostic biomarkers of metastatic melanoma. PLoS ONE.

[35] Bhalla S., Kaur H., Dhall A., Raghava G.P.S. (2019). Prediction and Analysis of Skin Cancer Progression using Genomics Profiles of Patients. Sci. Rep..

[36] Leick K.M., Rodriguez A.B., Melssen M.M., Benamar M., Lindsay R.S., Eki R., Du K.P., Parlak M., Abbas T., Engelhard V.H. (2019). The Barrier Molecules Junction Plakoglobin, Filaggrin, and Dystonin Play Roles in Melanoma Growth and Angiogenesis. Ann. Surg..

[37] Ricciardelli C., Lokman N.A., Pyragius C.E., Ween M.P., Macpherson A.M., Ruszkiewicz A., Hoffmann P., Oehler M.K. (2017). Keratin 5 overexpression is associated with serous ovarian cancer recurrence and chemotherapy resistance. Oncotarget.

[38] Vasca V., Vasca E., Freiman P., Marian D., Luce A., Mesolella M., Caraglia M., Ricciardiello F., Duminica T. (2014). Keratin 5 expression in squamocellular carcinoma of the head and neck. Oncol. Lett..

[39] Watt F.M. (1983). Involucrin and other markers of keratinocyte terminal differentiation. J. Investig. Dermatol..

[40] Azuma Y., Chou S.-C., Lininger R.A., Murphy B.J., Varia M.A., Raleigh J.A. (2003). Hypoxia and differentiation in squamous cell carcinomas of the uterine cervix: Pimonidazole and involucrin. Clin. Cancer Res..

[41] Tsuda H., Sakamaki C., Fukutomi T., Hirohashi S. (1997). Squamoid features and expression of involucrin in primary breast carcinoma associated with high histological grade, tumour cell necrosis and recurrence sites. Br. J. Cancer.

[42] Lan Y.-J., Chen H., Chen J.-Q., Lei Q.-H., Zheng M., Shao Z.-R. (2014). Immunolocalization of vimentin, keratin 17, Ki-67, involucrin, β-catenin and E-cadherin in cutaneous squamous cell carcinoma. Pathol. Oncol. Res..

[43] Yang L., Chen Y., Cui T., Knosel T., Zhang Q., Albring K.F., Huber O., Petersen I. (2012). Desmoplakin acts as a tumor suppressor by inhibition of the Wnt/beta-catenin signaling pathway in human lung cancer. Carcinogenesis.

[44] Nath A., Oak A., Chen K.Y., Li I., Splichal R.C., Portis J., Foster S., Walton S.P., Chan C. (2021). Palmitate-Induced IRE1–XBP1–ZEB Signaling Represses Desmoplakin Expression and Promotes Cancer Cell Migration. Mol. Cancer Res..

[45] Hodorogea A., Calinescu A., Antohe M., Balaban M., Nedelcu R.I., Turcu G., Ion D.A., Badarau I.A., Popescu C.M., Popescu R. (2019). Epithelial-Mesenchymal Transition in Skin Cancers: A Review. Anal. Cell. Pathol..

[46] Barrett T., Wilhite S.E., Ledoux P., Evangelista C., Kim I.F., Tomashevsky M., Marshall K.A., Phillippy K.H., Sherman P.M., Holko M. (2012). NCBI GEO: Archive for functional genomics data sets—Update. Nucleic Acids Res..

[47] Davis S., Meltzer P.S. (2007). GEOquery: A bridge between the Gene Expression Omnibus (GEO) and BioConductor. Bioinformatics.

[48] Ashburner M., Ball C.A., Blake J.A., Botstein D., Butler H., Cherry J.M., Davis A.P., Dolinski K., Dwight S.S., Eppig J.T. (2000). Gene ontology: Tool for the unification of biology. Nat. Genet..

[49] Sherman B.T., Lempicki R.A. (2009). Systematic and integrative analysis of large gene lists using DAVID bioinformatics resources. Nat. Protoc..

[50] Szklarczyk D., Franceschini A., Wyder S., Forslund K., Heller D., Huerta-Cepas J., Simonovic M., Roth A., Santos A., Tsafou K.P. (2015). STRING v10: Protein–protein interaction networks, integrated over the tree of life. Nucleic Acids Res..

[51] Shannon P., Marhiel A., Ozier O., Baliga N.S., Wang J.W., Ramage D., Amin D., Schwikowshi B., Ideker T. (2003). Cytoscape: A Software Enviroment for Intergrated Models of Biomolecular Interaction Networks. Genome Res..

[52] Li B., Shen W., Peng H., Li Y., Chen F., Zheng L., Xu J., Jia L. (2019). Fibronectin 1 promotes melanoma proliferation and metastasis by inhibiting apoptosis and regulating EMT. Onco Targets Ther..

[53] Li Y., Chen F., Shen W., Li B., Xiang R., Qu L., Zhang C., Li G., Xie H., Katanaev V.L. (2020). WDR74 induces nuclear beta-catenin accumulation and activates Wnt-responsive genes to promote lung cancer growth and metastasis. Cancer Lett..

[54] Shen W., Li Y., Li B., Zheng L., Xie X., Le J., Lu Y., Li T., Chen F., Jia L. (2019). Downregulation of KCTD12 contributes to melanoma stemness by modulating CD271. Cancer Biol. Med..

