# Supplementary material for: Identification of Core Genes and Pathways in Melanoma Metastasis via Bioinformatics Analysis
Source: Int J Mol Sci. 2022 Jan 12;23(2):794. doi: 10.3390/ijms23020794 (PMC8775799; doi:10.3390/ijms23020794)
Supplement: Supplementary file 1 [file ijms-23-00794-s001.zip › ijms-1506789-supplementary.pdf]

Supplementary materials

Identification of core genes and pathways in melanoma metastasis via bioinformatics analysis

Renjian Xie [1,2,3], Bifei Li [4], Lee Jia [4], and Yumei Li [1,5,*]

1 Key Laboratory of Prevention and treatment of cardiovascular and cerebrovascular diseases, Ministry of Education, Gannan Medical University, Ganzhou 341000, PR China

2 Key Laboratory of biomaterials and bio-fabrication in tissue engineering of Jiangxi Province, Ganzhou, 341000, China.

3 School of medical information engineering, Gannan Medical University, Ganzhou, 341000, China.

4 Institute of Oceanography, Minjiang University, Fuzhou, Fujian 350108, China

5 School of Basic Medicine, Gannan Medical University, Ganzhou, Jiangxi 341000, China.

* Correspondence: Yumei.Li@gmu.edu.cn (Y. L)

Supplementary Table S1. Differentially expressed genes (DEGs) between primary melanoma and metastatic melanoma.

**A. up-regulated DEGs**				
**ID**	**Gene symbol**	**Gene ID**	**Log2 (Fold change)**	**Adjusted *P* value**
205048_s_at	*PSPH*	5723	2.8486465	2.98E-10
209875_s_at	*SPP1*	6696	2.767764	1.99E-07
203819_s_at	*IGF2BP3*	10643	2.5198993	1.44E-08
202843_at	*DNAJB9*	4189	2.2987063	4.92E-08
209942_x_at	*MAGEA6*	4105	2.1640936	2.13E-03
209942_x_at	*MAGEA3*	4102	2.1640936	2.13E-03
202952_s_at	*ADAM12*	8038	2.1087045	2.97E-09
201890_at	*RRM2*	6241	1.9764716	1.94E-06
204627_s_at	*ITGB3*	3690	1.9427608	2.67E-05
202533_s_at	*DHFR*	1719	1.8997797	1.24E-06
206859_s_at	*PAEP*	5047	1.8672555	6.61E-03
203213_at	*CDK1*	983	1.8363552	1.61E-06
208358_s_at	*UGT8*	7368	1.8051681	6.62E-06
218748_s_at	*EXOC5*	10640	1.7962246	3.13E-06
219555_s_at	*CENPN*	55839	1.7951241	3.07E-05
213906_at	*MYBL1*	4603	1.7812887	9.34E-06

207144_s_at	*CITED1*	4435	1.7730732	7.27E-04
204361_s_at	*SKAP2*	8935	1.7615603	8.99E-07
206392_s_at	*RARRES1*	5918	1.7561882	2.70E-04
205884_at	*ITGA4*	3676	1.7478544	8.49E-05
211708_s_at	*SCD*	6319	1.7442142	6.38E-06
204107_at	*NFYA*	4800	1.6894207	7.78E-07
205860_x_at	*FOLH1B*	219595	1.6706834	2.34E-04
205860_x_at	*FOLH1*	2346	1.6706834	2.34E-04
203362_s_at	*MAD2L1*	4085	1.6694282	4.75E-07
202620_s_at	*PLOD2*	5352	1.6684364	1.07E-05
220651_s_at	*MCM10*	55388	1.6623529	2.37E-07
204362_at	*SKAP2*	8935	1.6429543	6.57E-08
211205_x_at	*PIP5K1A*	8394	1.6374452	1.23E-05
209642_at	*BUB1*	699	1.6227922	4.12E-09
220789_s_at	*SNORA5B///TBRG4*	677795///9238	1.6125486	5.89E-06
205395_s_at	*MRE11A*	4361	1.6105592	2.80E-05
204051_s_at	*SFRP4*	6424	1.604949	4.03E-07
213999_at	*YIPF4*	84272	1.6044305	8.55E-09
221781_s_at	*DNAJC10*	54431	1.6035957	1.24E-05
221520_s_at	*CDCA8*	55143	1.6010758	1.98E-05
204641_at	*NEK2*	4751	1.6003376	2.15E-04
221703_at	*BRIP1*	83990	1.5847153	1.02E-05
211574_s_at	*CD46*	4179	1.5845995	1.60E-05
204357_s_at	*LIMK1*	3984	1.5836684	2.12E-05
201481_s_at	*PYGB*	5834	1.5778202	2.20E-03
218741_at	*CENPM*	79019	1.5631817	9.01E-06
201661_s_at	*ACSL3*	2181	1.5607804	2.95E-08
205194_at	*PSPH*	5723	1.559975	4.44E-08
203165_s_at	*SLC33A1*	9197	1.5588155	8.84E-06
212396_s_at	*EMC1*	23065	1.5547863	3.93E-04
219918_s_at	*ASPM*	259266	1.5533115	9.39E-05
210447_at	*GLUD2*	2747	1.5475927	9.34E-06
210148_at	*HIPK3*	10114	1.5473892	5.59E-06
207739_s_at	*GAGE12D///GAGE8///GAGE12F///GAGE2A///GAGE12H///GAGE12E///GAGE12B///GAGE12C///GAGE2D///GAGE12J///GAGE12G///GAGE13///GAGE2B///GAGE2E///*	100132399///100101629///100008586///729447///729442///729431///729428///729422///729408///729396///645073///6450	1.5445789	4.32E-02

	GAGE12I///GAGE7///GAGE6///GAGE5///GAGE4///GAGE3///GAGE2C///GAGE1	51///645037///26749///26748///2579///2578///2577///2576///2575///2574///2543		
218663_at	*NCAPG*	64151	1.5357071	3.69E-05
201795_at	*LBR*	3930	1.5300583	2.35E-06
220840_s_at	*C1orf112*	55732	1.5291092	2.22E-05
204344_s_at	*SEC23A*	10484	1.5278086	6.21E-06
214008_at	*TWF1*	5756	1.5268933	5.94E-05
202558_s_at	*HSPA13*	6782	1.5255924	4.64E-09
210559_s_at	*CDK1*	983	1.5213109	3.04E-07
206205_at	*MPHOSPH9*	10198	1.5207669	4.73E-04
216392_s_at	*SEC23IP*	11196	1.5188886	5.03E-07
201042_at	*TGM2*	7052	1.5018492	5.54E-12

B. down-regulated DEGs

ID	Gene symbol	Gene ID	Log2 (Fold change)	Adjusted *P* value
205916_at	*S100A7*	6278	9.04E-20	-8.6805712
209351_at	*KRT14*	3861	6.10E-17	-8.6624659
209800_at	*KRT16*	3868	1.73E-20	-8.0258657
213796_at	*SPRR1A*	6698	2.56E-19	-7.8002823
209125_at	*KRT6A*	3853	1.06E-18	-6.8262439
205157_s_at	*KRT17*	3872	1.41E-19	-6.7016996
205157_s_at	*JUP*	3728	1.41E-19	-6.7016996
201820_at	*KRT5*	3852	1.78E-17	-6.6593224
214580_x_at	*KRT6C*	2868873	1.35E-18	-6.4903185
214580_x_at	*KRT6B*	3854	1.35E-18	-6.4903185
207720_at	*LOR*	4014	1.94E-16	-6.4902197
209260_at	*SFN*	2810	6.29E-19	-6.3583246
206400_at	*LGALS7B*	653499	4.02E-15	-6.2382627
206400_at	*LGALS7*	3963	4.02E-15	-6.2382627
221854_at	*PKP1*	5317	1.14E-17	-6.0861137
214370_at	*S100A8*	6279	2.56E-19	-6.0784181
215704_at	*FLG*	2312	4.24E-14	-5.9593454
205900_at	*KRT1*	3848	7.05E-17	-5.9046423
209720_s_at	*SERPINB3*	6317	2.18E-16	-5.8325273
220414_at	*CALML5*	51806	4.94E-18	-5.7479842
204734_at	*KRT15*	3866	1.61E-15	-5.7268871
205064_at	*SPRR1B*	6699	2.56E-19	-5.684485
213680_at	*KRT6B*	3854	1.83E-19	-5.6662477
203691_at	*PI3*	5266	3.71E-14	-5.6644747

206276_at	*LY6D*	8581	2.72E-17	-5.5759145
212236_x_at	*KRT17*	3872	6.04E-21	-5.5634888
204855_at	*SERPINB5*	5268	1.78E-17	-5.3751852
202917_s_at	*S100A8*	6279	6.03E-16	-5.2585042
202504_at	*TRIM29*	23650	3.61E-17	-5.137469
206642_at	*DSG1*	1828	1.02E-14	-4.9765378
204268_at	*S100A2*	6273	7.42E-19	-4.9563942
211002_s_at	*TRIM29*	23650	5.34E-18	-4.8606567
204952_at	*LYPD3*	27076	1.01E-18	-4.7818787
208539_x_at	*SPRR2B*	6701	8.42E-13	-4.7578044
202286_s_at	*TACSTD2*	4070	2.37E-16	-4.7499509
205185_at	*SPINK5*	11005	2.92E-14	-4.4718565
33323_r_at	*SFN*	2810	9.04E-20	-4.4288895
217528_at	*CLCA2*	9635	7.95E-14	-4.3463473
206192_at	*CDSN*	1041	4.02E-15	-4.276411
203407_at	*PPL*	5493	3.92E-12	-4.2529182
203535_at	*S100A9*	6280	3.33E-15	-4.241793
214549_x_at	*SPRR1A*	6698	9.92E-18	-4.1857849
207381_at	*ALOX12B*	242	1.03E-16	-4.1194521
39248_at	*AQP3*	360	4.50E-17	-4.1152733
210020_x_at	*CALML3*	810	1.03E-16	-4.0573585
206193_s_at	*CDSN*	1041	2.90E-11	-4.0367733
200606_at	*DSP*	1832	5.15E-15	-4.035886
207324_s_at	*DSC1*	1823	7.95E-14	-4.0305464
204379_s_at	*FGFR3*	2261	2.32E-12	-4.0148948
206595_at	*CST6*	1474	3.13E-11	-4.0072647
211906_s_at	*SERPINB4*	6318	8.41E-11	-4.0064569
206164_at	*CLCA2*	9635	6.22E-15	-3.9902282
218186_at	*RAB25*	57111	2.94E-14	-3.9820158
33322_i_at	*SFN*	2810	2.42E-20	-3.9742324
204636_at	*COL17A1*	1308	3.86E-14	-3.9562656
209792_s_at	*KLK10*	5655	6.22E-15	-3.8582876
220724_at	*CWH43*	80157	2.14E-13	-3.8530661
204971_at	*CSTA*	1475	1.26E-11	-3.8136783
219995_s_at	*ZNF750*	79755	2.14E-11	-3.8125426
220016_at	*AHNAK*	79026	1.31E-20	-3.7952858
201015_s_at	*JUP*	3728	8.73E-16	-3.7916081
219597_s_at	*DUOX1*	53905	3.38E-14	-3.7408712
205694_at	*TYRP1*	7306	1.93E-06	-3.7074093
210084_x_at	*TPSAB1*	7177	3.23E-12	-3.6929214
204503_at	*EVPL*	2125	1.45E-18	-3.6910469
220620_at	*CRCT1*	54544	1.71E-11	-3.6640812
220013_at	*EPHX3*	79852	2.67E-13	-3.6276793
207710_at	*LCE2B*	26239	4.08E-16	-3.6172844

204750_s_at	*DSC2*	1824	1.27E-13	-3.536802
41469_at	*PI3*	5266	1.35E-11	-3.5299417
205470_s_at	*KLK11*	11012	7.32E-13	-3.5004835
219529_at	*CLIC3*	9022	4.26E-10	-3.4195677
205627_at	*CDA*	978	1.07E-13	-3.3913501
218002_s_at	*CXCL14*	9547	1.22E-10	-3.3683316
209719_x_at	*SERPINB3*	6317	5.00E-14	-3.3649877
204870_s_at	*PCSK2*	5126	1.32E-08	-3.3536241
206033_s_at	*DSC3*	1825	5.37E-16	-3.351468
203453_at	*SCNN1A*	6337	4.15E-13	-3.3167762
206122_at	*SOX15*	6665	1.40E-17	-3.3048995
205363_at	*BBOX1*	8424	3.31E-11	-3.300847
206166_s_at	*CLCA2*	9635	5.88E-14	-3.28285
266_s_at	*CD24*	100133941	6.16E-08	-3.2820212
204508_s_at	*CA12*	771	1.43E-14	-3.2803932
203699_s_at	*DIO2*	1734	7.27E-12	-3.2627859
218657_at	*RAPGEFL1*	51195	4.76E-13	-3.2538698
206023_at	*NMU*	10874	1.22E-11	-3.2435618
206008_at	*TGM1*	7051	4.94E-12	-3.2165395
203256_at	*CDH3*	1001	6.57E-07	-3.1474049
209771_x_at	*CD24*	100133941	2.07E-08	-3.1404535
214451_at	*TFAP2B*	7021	5.49E-07	-3.1131219
207109_at	*POU2F3*	25833	5.29E-10	-3.054846
213287_s_at	*KRT10*	3858	2.24E-17	-3.0395739
219476_at	*C1orf116*	79098	1.67E-15	-3.021505
210413_x_at	*SERPINB4///SERPINB3*	6318///6317	2.32E-12	-3.017178
219554_at	*RHCG*	51458	9.86E-12	-3.0171674
203726_s_at	*LAMA3*	3909	1.27E-11	-3.0125491
214599_at	*IVL*	3713	3.17E-11	-3.0071085
217744_s_at	*PERP*	64065	8.28E-12	-2.9816277
207023_x_at	*KRT10*	3858	8.02E-19	-2.9815819
220318_at	*EPN3*	55040	4.69E-12	-2.9128439
220225_at	*IRX4*	50805	1.70E-13	-2.9118487
39249_at	*AQP3*	360	6.66E-13	-2.9118295
206165_s_at	*CLCA2*	9635	4.62E-11	-2.9070565
217023_x_at	*TPSB2///TPSAB1*	64499///7177	2.49E-10	-2.8964723
206032_at	*DSC3*	1825	2.57E-14	-2.883662
209885_at	*RHOD*	29984	5.57E-15	-2.8731996
205490_x_at	*GJB3*	2707	7.76E-16	-2.8711276
201983_s_at	*EGFR*	1956	5.16E-12	-2.8563975
207908_at	*KRT2*	3849	3.01E-12	-2.8404687
203638_s_at	*FGFR2*	2263	1.03E-10	-2.8399851
218876_at	*TPPP3*	51673	1.28E-11	-2.8365198

205595_at	*DSG3*	1830	1.13E-13	-2.8307073
218677_at	*S100A14*	57402	1.09E-16	-2.825635
216641_s_at	*LAD1*	3898	3.03E-13	-2.8072051
204136_at	*COL7A1*	1294	1.07E-13	-2.793215
219756_s_at	*POF1B*	79983	1.62E-12	-2.7785138
222383_s_at	*ALOXE3*	59344	2.70E-14	-2.7772515
205403_at	*IL1R2*	7850	2.17E-09	-2.7683574
206177_s_at	*ARG1*	383	4.85E-13	-2.7635419
202575_at	*CRABP2*	1382	2.72E-17	-2.7600286
210633_x_at	*KRT10*	3858	7.14E-17	-2.7462344
216379_x_at	*CD24*	100133941	2.42E-08	-2.7382421
206884_s_at	*SCEL*	8796	1.86E-08	-2.7325365
202826_at	*SPINT1*	6692	4.66E-12	-2.7152915
204351_at	*S100P*	6286	9.51E-12	-2.710734
210132_at	*EFNA3*	1944	1.17E-08	-2.7070548
201286_at	*SDC1*	6382	7.28E-13	-2.7039637
211748_x_at	*PTGDS*	5730	7.01E-08	-2.6961264
204990_s_at	*ITGB4*	3691	1.87E-15	-2.6885092
205778_at	*KLK7*	5650	1.77E-08	-2.6770003
207114_at	*LY6G6C*	80740	2.37E-13	-2.6722758
203074_at	*ANXA8L1///ANXA8*	728113///653145	1.53E-16	-2.6664954
205987_at	*CD1C*	911	6.50E-07	-2.652161
204455_at	*DST*	667	3.47E-12	-2.6504825
205014_at	*FGFBP1*	9982	5.94E-17	-2.6403885
206385_s_at	*ANK3*	288	1.20E-06	-2.6396615
206421_s_at	*SERPINB7*	8710	1.28E-09	-2.6261121
209212_s_at	*KLF5*	688	4.02E-15	-2.6073538
203000_at	*STMN2*	11075	8.04E-09	-2.6009229
204942_s_at	*ALDH3B2*	222	8.16E-09	-2.5786928
219936_s_at	*GPR87*	53836	9.99E-09	-2.5769131
209772_s_at	*CD24*	100133941	1.62E-07	-2.5758752
206482_at	*PTK6*	5753	6.76E-08	-2.5607131
213451_x_at	*TNXB///TNXA*	7148///7146	5.55E-09	-2.5574928
209074_s_at	*FAM107A*	11170	6.00E-10	-2.5516444
207065_at	*KRT75*	9119	6.38E-14	-2.5512973
219998_at	*LGALSL*	29094	2.32E-09	-2.5475041
205713_s_at	*COMP*	1311	3.26E-05	-2.5245608
216333_x_at	*TNXB///TNXA*	7148///7146	1.44E-08	-2.5242808
202890_at	*MAP7*	9053	1.27E-06	-2.5242109
215382_x_at	*TPSAB1*	7177	2.95E-08	-2.5123857
212187_x_at	*PTGDS*	5730	8.00E-08	-2.5026387
201131_s_at	*CDH1*	999	1.30E-03	-2.4983286
210397_at	*DEFB1*	1672	2.04E-11	-2.495886

208606_s_at	*WNT4*	54361	3.33E-11	-2.4879943
206093_x_at	*TNXB///TNXA*	7148///7146	1.70E-07	-2.4840643
218963_s_at	*KRT23*	25984	2.44E-10	-2.4712331
217546_at	*MT1M*	4499	4.26E-06	-2.4706309
205683_x_at	*TPSAB1*	7177	9.14E-11	-2.4572989
219630_at	*PDZK1IP1*	10158	2.71E-09	-2.4360842
209309_at	*AZGP1*	563	4.90E-09	-2.4355791
207134_x_at	*TPSB2*	64499	1.44E-09	-2.4347818
209016_s_at	*KRT7*	3855	3.49E-09	-2.4343345
206643_at	*HAL*	3034	1.62E-09	-2.4324554
217272_s_at	*SERPINB13*	5275	4.31E-12	-2.4300197
203021_at	*SLPI*	6590	1.95E-10	-2.4271444
209863_s_at	*TP63*	8626	3.05E-14	-2.4244451
222242_s_at	*KLK5*	25818	1.07E-13	-2.4067452
206561_s_at	*AKR1B10*	57016	1.47E-10	-2.3855124
205623_at	*ALDH3A1*	218	1.84E-07	-2.385182
220412_x_at	*KCNK7*	10089	3.06E-13	-2.3684969
206156_at	*GJB5*	2709	1.09E-09	-2.3656748
206125_s_at	*KLK8*	11202	5.66E-10	-2.3538107
213369_at	*CDHR1*	92211	1.07E-07	-2.3210858
207955_at	*CCL27*	10850	9.72E-09	-2.3094896
203240_at	*FCGBP*	8857	9.70E-08	-2.3036607
211663_x_at	*PTGDS*	5730	3.04E-08	-2.3004737
211372_s_at	*IL1R2*	7850	7.38E-08	-2.2860236
91826_at	*EPS8L1*	54869	1.30E-12	-2.2844516
218796_at	*FERMT1*	55612	1.66E-08	-2.2792373
209873_s_at	*PKP3*	11187	2.54E-13	-2.2677432
211043_s_at	*CLTB*	1212	4.02E-15	-2.2649962
220635_at	*PSORS1C2*	170680	3.76E-09	-2.2628605
205767_at	*EREG*	2069	1.26E-07	-2.251171
214536_at	*SLURP1*	57152	4.21E-09	-2.2483346
219410_at	*TMEM45A*	55076	1.16E-08	-2.2449545
210086_at	*HR*	55806	7.98E-08	-2.2446327
208345_s_at	*POU3F1*	5453	4.90E-09	-2.2393678
211597_s_at	*HOPX*	84525	9.25E-08	-2.2316578
205081_at	*CRIP1*	1396	4.84E-08	-2.2314247
203571_s_at	*ADIRF*	10974	7.41E-08	-2.2276277
212531_at	*LCN2*	3934	3.72E-07	-2.2213316
211734_s_at	*FCER1A*	2205	2.16E-10	-2.2199389
216474_x_at	*TPSB2///TPSAB1*	64499///7177	6.15E-09	-2.2160978
210715_s_at	*SPINT2*	10653	6.89E-07	-2.2109899
208651_x_at	*CD24*	100133941	6.17E-06	-2.2093801
208190_s_at	*LSR*	51599	6.94E-07	-2.2006625
213217_at	*ADCY2*	108	1.65E-04	-2.198827

206149_at	*CHP2*	63928	8.85E-11	-2.1896603
212242_at	*TUBA4A*	7277	1.10E-13	-2.1877667
219411_at	*ELMO3*	79767	5.04E-08	-2.1865741
220723_s_at	*CWH43*	80157	1.96E-08	-2.1835763
212992_at	*AHNAK2*	113146	6.82E-06	-2.1810885
206315_at	*CRLF1*	9244	5.86E-09	-2.1733692
203215_s_at	*MYO6*	4646	4.44E-08	-2.1731506
214164_x_at	*CA12*	771	2.85E-13	-2.1730391
218484_at	*NDUFA4L2*	56901	7.18E-11	-2.1630865
215536_at	*HLA-DQB2*	3120	4.52E-10	-2.1583475
221841_s_at	*KLF4*	9314	4.11E-13	-2.1539956
213506_at	*F2RL1*	2150	1.02E-06	-2.1477137
203287_at	*LAD1*	3898	2.71E-15	-2.1447424
203913_s_at	*HPGD*	3248	5.58E-04	-2.1400112
203798_s_at	*VSNL1*	7447	4.41E-07	-2.1393393
222223_s_at	*IL36RN*	26525	5.54E-12	-2.1280979
202712_s_at	*CKMT1A///CKMT1B*	548596///1159	8.26E-11	-2.1095058
204475_at	*MMP1*	4312	7.83E-04	-2.1085895
207254_at	*SLC15A1*	6564	6.02E-06	-2.107787
220403_s_at	*TP53AIP1*	63970	1.59E-10	-2.1031429
219825_at	*CYP26B1*	56603	1.06E-07	-2.0997985
213456_at	*SOSTDC1*	25928	1.25E-04	-2.098227
203963_at	*CA12*	771	8.75E-12	-2.089371
212713_at	*MFAP4*	4239	1.22E-05	-2.0724543
206114_at	*EPHA4*	2043	2.79E-04	-2.0722886
216512_s_at	*DCT*	1638	4.85E-03	-2.068582
214071_at	*GNAL*	2774	6.45E-05	-2.0529535
205337_at	*DCT*	1638	6.28E-03	-2.0482862
205382_s_at	*CFD*	1675	4.84E-05	-2.0475272
215395_x_at	*PRSS3P2*	154754	2.49E-07	-2.0410968
213182_x_at	*CDKN1C*	1028	1.31E-06	-2.0394797
209373_at	*MALL*	7851	5.54E-12	-2.0354859
216905_s_at	*ST14*	6768	7.29E-07	-2.0343281
204989_s_at	*ITGB4*	3691	4.12E-09	-2.0336116
205338_s_at	*DCT*	1638	7.70E-04	-2.0310534
221950_at	*EMX2*	2018	4.48E-06	-2.0286399
205200_at	*EXOSC7///CLEC3B*	23016///7123	1.28E-07	-2.0272758
219850_s_at	*EHF*	26298	1.21E-06	-2.0270797
207463_x_at	*PRSS3*	5646	1.13E-11	-2.0239573
210072_at	*CCL19*	6363	9.51E-05	-2.0196369
216918_s_at	*DST*	667	3.77E-10	-2.0149396
215014_at	*KCND3*	3752	1.41E-04	-2.0149166

201667_at	*GJA1*	2697	1.42E-08	-2.0145617
209211_at	*KLF5*	688	4.27E-11	-1.9988046
213496_at	*PLPPR4*	9890	1.91E-10	-1.9985322
205709_s_at	*CDS1*	1040	1.37E-06	-1.9969662
220289_s_at	*AIM1L*	55057	1.77E-09	-1.994082
211737_x_at	*PTN*	5764	3.61E-08	-1.9911437
202489_s_at	*FXYD3*	5349	1.40E-03	-1.990481
202525_at	*PRSS8*	5652	3.98E-12	-1.9792305
209602_s_at	*GATA3*	2625	2.57E-05	-1.977405
220266_s_at	*KLF4*	9314	4.11E-11	-1.9488214
203747_at	*AQP3*	360	2.12E-08	-1.9341626
201497_x_at	*MYH11*	4629	2.54E-04	-1.9279265
221665_s_at	*EPS8L1*	54869	9.31E-11	-1.9276059
208335_s_at	*ACKR1*	2532	3.29E-04	-1.9257637
32128_at	*CCL18*	6362	1.43E-04	-1.88492
205759_s_at	*SULT2B1*	6820	7.15E-15	-1.8842523
202345_s_at	*FABP5*	2171	9.38E-09	-1.8840553
201428_at	*CLDN4*	1364	1.15E-07	-1.8832513
210128_s_at	*LTB4R*	1241	1.85E-10	-1.870204
209871_s_at	*APBA2*	321	2.16E-06	-1.8694336
221900_at	*COL8A2*	1296	6.78E-06	-1.8590572
215784_at	*CD1E*	913	1.15E-04	-1.851359
215465_at	*ABCA12*	26154	1.29E-06	-1.8321443
213071_at	*DPT*	1805	2.52E-05	-1.8297502
201287_s_at	*SDC1*	6382	9.40E-08	-1.8227864
220066_at	*NOD2*	64127	6.81E-06	-1.819631
205220_at	*HCAR3*	8843	1.60E-06	-1.8182571
220664_at	*SPRR2C*	6702	6.75E-06	-1.8157332
219534_x_at	*CDKN1C*	1028	1.85E-06	-1.81338
219919_s_at	*SSH3*	54961	5.76E-07	-1.8129198
205653_at	*CTSG*	1511	1.16E-10	-1.8088783
203886_s_at	*FBLN2*	2199	9.21E-06	-1.8065528
211549_s_at	*HPGD*	3248	2.27E-04	-1.8037467
215867_x_at	*CA12*	771	3.92E-12	-1.7982853
218736_s_at	*PALMD*	54873	1.92E-09	-1.7933867
209570_s_at	*NSG1*	27065	8.90E-04	-1.7928155
216258_s_at	*SERPINB13*	5275	3.49E-05	-1.7881956
205155_s_at	*SPTBN2*	6712	7.88E-07	-1.7874676
204733_at	*KLK6*	5653	2.73E-05	-1.785394
203001_s_at	*STMN2*	11075	1.01E-06	-1.7850268
203126_at	*IMPA2*	3613	4.64E-09	-1.7825051
219369_s_at	*OTUB2*	78990	1.03E-07	-1.7783453
207741_x_at	*TPSAB1*	7177	2.66E-10	-1.7660794
205349_at	*GNA15*	2769	4.75E-08	-1.7659377

219395_at	*MIR6773///ESRP2*	102466194///80004	2.62E-09	-1.7548624
205870_at	*BDKRB2*	624	6.76E-09	-1.7519809
209270_at	*LAMB3*	3914	9.26E-12	-1.7508563
201348_at	*GPX3*	2878	9.25E-08	-1.7335694
204941_s_at	*ALDH3B2*	222	1.82E-04	-1.7301151
219909_at	*MMP28*	79148	1.20E-06	-1.7296068
219197_s_at	*SCUBE2*	57758	4.31E-05	-1.7155089
202177_at	*GAS6*	2621	8.84E-09	-1.7154573
213421_x_at	*PRSS3*	5646	1.23E-11	-1.7138168
218451_at	*CDCP1*	64866	1.28E-06	-1.7135438
219010_at	*C1orf106*	55765	5.83E-06	-1.7115305
200636_s_at	*PTPRF*	5792	2.12E-07	-1.7069053
211548_s_at	*HPGD*	3248	1.34E-03	-1.705238
217087_at	*C1orf68*	100129271	1.18E-05	-1.7029161
216470_x_at	*PRSS2*	5645	6.94E-08	-1.6972597
208502_s_at	*PITX1*	5307	2.42E-05	-1.6910884
60474_at	*FERMT1*	55612	1.07E-07	-1.6906976
214247_s_at	*DKK3*	27122	7.98E-07	-1.6896213
206378_at	*SCGB2A2*	4250	3.09E-03	-1.68425
206655_s_at	*SEPT5-GP1BB///SEPT5///GP1BB*	100526833///5413///2812	5.62E-06	-1.6780336
209604_s_at	*GATA3*	2625	4.54E-07	-1.6761567
205172_x_at	*CLTB*	1212	1.18E-14	-1.6744601
202597_at	*IRF6*	3664	1.16E-10	-1.6721234
208168_s_at	*CHIT1*	1118	1.06E-03	-1.6710695
202831_at	*GPX2*	2877	4.71E-08	-1.6611658
209301_at	*CA2*	760	1.55E-05	-1.6563209
221795_at	*NTRK2*	4915	1.06E-05	-1.656096
207935_s_at	*KRT13*	3860	6.23E-05	-1.6517676
219407_s_at	*LAMC3*	10319	4.69E-05	-1.6499985
207861_at	*CCL22*	6367	4.97E-07	-1.6384537
216935_at	*LINC00302*	388699	8.54E-06	-1.6384486
204753_s_at	*HLF*	3131	8.44E-06	-1.6318942
200635_s_at	*PTPRF*	5792	8.08E-06	-1.6308437
206284_x_at	*CLTB*	1212	1.48E-17	-1.6198423
211361_s_at	*SERPINB13*	5275	1.08E-05	-1.6171737
40016_g_at	*MAST4*	375449	8.18E-14	-1.6132721
200965_s_at	*ABLIM1*	3983	4.76E-09	-1.6120693
214382_at	*UNC93A*	54346	2.42E-05	-1.608113
203700_s_at	*DIO2*	1734	1.47E-10	-1.5922859
220638_s_at	*CBLC*	23624	1.41E-06	-1.5816272
202196_s_at	*DKK3*	27122	3.85E-05	-1.5744099
201425_at	*ALDH2*	217	8.21E-07	-1.5624204

202935_s_at	*SOX9*	6662	3.22E-05	-1.5624053
204614_at	*SERPINB2*	5055	6.72E-07	-1.5623464
220994_s_at	*STXBP6*	29091	4.55E-04	-1.5529484
219729_at	*PRRX2*	51450	9.14E-11	-1.551813
218995_s_at	*EDN1*	1906	6.76E-06	-1.5508053
206799_at	*SCGB1D2*	10647	1.40E-03	-1.548644
218180_s_at	*EPS8L2*	64787	4.47E-08	-1.5476125
203961_at	*NEBL*	10529	5.01E-05	-1.5441634
213425_at	*WNT5A*	7474	1.72E-04	-1.5439017
216894_x_at	*CDKN1C*	1028	4.27E-05	-1.5434632
219327_s_at	*GPRC5C*	55890	1.19E-05	-1.5418059
218644_at	*PLEK2*	26499	4.38E-06	-1.5385353
219532_at	*ELOVL4*	6785	6.82E-04	-1.5373199
205422_s_at	*ITGBL1*	9358	8.09E-04	-1.5370819
213693_s_at	*MUC1*	4582	7.78E-05	-1.5349756
202267_at	*LAMC2*	3918	1.32E-08	-1.5333349
219764_at	*FZD10*	11211	1.13E-06	-1.5330966
207961_x_at	*MYH11*	4629	3.48E-04	-1.5321779
209465_x_at	*PTN*	5764	5.01E-05	-1.5319359
220780_at	*PLA2G3*	50487	7.45E-07	-1.52941
202728_s_at	*LTBP1*	4052	3.12E-06	-1.5292271
204363_at	*F3*	2152	1.88E-04	-1.5267383
211215_x_at	*DIO2*	1734	1.07E-09	-1.5244169
209691_s_at	*DOK4*	55715	9.81E-06	-1.5195238
209603_at	*GATA3*	2625	6.96E-05	-1.5186661
205212_s_at	*ACAP1*	9744	6.90E-04	-1.5161546
221295_at	*CIDEA*	1149	8.25E-07	-1.5127725
206539_s_at	*CYP4F12*	66002	8.90E-06	-1.5126249
208609_s_at	*TNXB///TNXA*	7148///7146	5.72E-06	-1.5125123
200637_s_at	*PTPRF*	5792	6.38E-05	-1.5124306
204606_at	*CCL21*	6366	2.11E-02	-1.5101459
218980_at	*FHOD3*	80206	1.29E-03	-1.5054611
204777_s_at	*MAL*	4118	1.54E-05	-1.5054219
217312_s_at	*COL7A1*	1294	2.64E-07	-1.5039241
210619_s_at	*HYAL1*	3373	9.50E-07	-1.5034959
219695_at	*SMPD3*	55512	2.57E-06	-1.5031308
207008_at	*CXCR2*	3579	1.58E-04	-1.5013707
209126_x_at	*KRT6B*	3854	8.67E-19	-1.5009977
209156_s_at	*COL6A2*	1292	3.29E-09	-1.5008512

Supplementary Table S2. GO analysis of DEGs associated with melanoma metastasis.(FDR: false discovery rate)

Category	Term	Count	*P* Value	FDR
**A. Up-regulated DEGs**				
**Category**	**Term**	**Count**	***P* Value**	**FDR**
GOTERM_BP_FAT	GO:0000819~sister chromatid segregation	7	2.91E-05	4.90E-48
GOTERM_BP_FAT	GO:1903047~mitotic cell cycle process	11	1.04E-04	1.76E-37
GOTERM_BP_FAT	GO:0098813~nuclear chromosome segregation	7	1.15E-04	1.90E-32
GOTERM_BP_FAT	GO:0007067~mitotic nuclear division	8	1.44E-04	2.40E-28
GOTERM_BP_FAT	GO:0022402~cell cycle process	13	1.87E-04	3.13E-24
GOTERM_CC_FAT	GO:0000777~condensed chromosome kinetochore	5	3.09E-04	3.81E-15
GOTERM_CC_FAT	GO:0000775~chromosome, centromeric region	6	3.14E-04	3.80E-15
GOTERM_CC_FAT	GO:0000779~condensed chromosome, centromeric region	5	4.26E-04	5.24E-11
GOTERM_CC_FAT	GO:0000793~condensed chromosome	6	5.10E-04	6.26E-09
GOTERM_CC_FAT	GO:0098687~chromosomal region	7	7.33E-04	9.00E-07
GOTERM_MF_FAT	GO:0036094~small molecule binding	16	0.001218847	1.59E-05
GOTERM_MF_FAT	GO:0032559~adenyl ribonucleotide binding	12	0.001322438	1.37E-05
GOTERM_MF_FAT	GO:0030554~adenyl nucleotide binding	12	0.001402442	9.33E-04
GOTERM_MF_FAT	GO:0005524~ATP binding	11	0.003900765	6.93E-04
GOTERM_MF_FAT	GO:0000166~nucleotide binding	14	0.005471503	6.83E-04
**B. Down-regulated DEGs**				
**Category**	**Term**	**Count**	***P* Value**	**FDR**
GOTERM_BP_FAT	GO:0008544~epidermis development	55	7.24E-42	1.36E-38
GOTERM_BP_FAT	GO:0043588~skin development	43	2.57E-32	4.82E-29
GOTERM_BP_FAT	GO:0030216~keratinocyte differentiation	30	1.12E-26	2.09E-23
GOTERM_BP_FAT	GO:0009913~epidermal cell differentiation	33	6.61E-25	1.24E-21
GOTERM_BP_FAT	GO:0030855~epithelial cell differentiation	50	4.45E-23	8.35E-20
GOTERM_CC_FAT	GO:0005576~extracellular region	165	2.67E-27	3.67E-24

GOTERM_CC_FAT	GO:0044421~extracellular region part	149	9.38E-27	1.29E-23
GOTERM_CC_FAT	GO:0070062~extracellular exosome	110	2.61E-18	3.57E-15
GOTERM_CC_FAT	GO:1903561~extracellular vesicle	110	3.80E-18	5.21E-15
GOTERM_CC_FAT	GO:0043230~extracellular organelle	110	3.90E-18	5.35E-15
GOTERM_MF_FAT	GO:0005198~structural molecule activity	46	6.04E-14	9.02E-11
GOTERM_MF_FAT	GO:0004866~endopeptidase inhibitor activity	16	1.41E-07	2.10E-04
GOTERM_MF_FAT	GO:0005200~structural constituent of cytoskeleton	13	2.01E-07	3.00E-04
GOTERM_MF_FAT	GO:0061135~endopeptidase regulator activity	16	2.20E-07	3.28E-04
GOTERM_MF_FAT	GO:0030414~peptidase inhibitor activity	16	2.72E-07	4.07E-04
